# Effects of Feline Herpesvirus Type 1 Vaccination on Clinical Disease, Viral Shedding, and Post-Vaccination Reactions: A Systematic Review and Exploratory Meta-Analysis

**DOI:** 10.3390/v18101115

**Published:** 2026-10-08

**Authors:** Zhen Lei, Chang Liu, Zhihua Yang, Xianzheng Yin, Wuchao Zhang, Jiangwei Song

**Affiliations:** 1 Beijing Key Laboratory for Prevention and Control of Infectious Diseases in Livestock and Poultry, Institute of Animal Husbandry and Veterinary Medicine, Beijing Academy of Agriculture and Forestry Sciences, Beijing 100097, China; 2 College of Veterinary Medicine, Hebei Agricultural University, Baoding 071000, China; 3 College of Life Science, Hebei University, Baoding 071002, China

**Keywords:** feline herpesvirus type 1, vaccine, clinical disease, viral shedding, post-vaccination reactions, latency, systematic review, exploratory meta-analysis

## Abstract

Feline herpesvirus type 1 (FHV-1) causes respiratory and ocular disease in cats and establishes lifelong latency, but the extent to which vaccination modifies clinical disease, viral shedding, and latency-related outcomes remains uncertain. We systematically reviewed controlled in vivo studies of FHV-1 vaccination in cats, searching PubMed, Web of Science, and the CNKI from inception to 20 May 2026. Risk of bias was assessed with the SYRCLE tool and certainty for core outcomes with the GRADE approach. Exploratory random-effects meta-analyses were undertaken only when outcomes were sufficiently comparable. Of 327 records identified, 23 reports describing 20 independent animal studies (25 cohorts) were included. Fifteen reports evaluated shedding or viral load, 20 assessed immunogenicity, and one companion report examined latency and reactivation. In two comparable challenge studies involving 17 vaccinated and nine control cats, vaccination was associated with fewer post-challenge clinical signs (risk ratio [RR] = 0.64; 95% confidence interval [CI] 0.41 to 0.99). In one monovalent modified-live vaccine study, cumulative clinical scores were lower after vaccination (mean difference [MD] = −36.00; 95% CI −57.25 to −14.75), with Hedges’ *g* = −1.90 (95% CI −3.52 to −0.27). Estimates for fever, disease-related removal, and post-vaccination sneezing were imprecise. Most virological studies reported lower shedding or viral loads after vaccination, but heterogeneous assays, outcome definitions, and observation windows precluded robust pooling. Antibody responses could not be directly equated with clinical protection. Overall, FHV-1 vaccination may mitigate post-challenge clinical disease, whereas evidence for effects on shedding, post-vaccination reactions, latency, and reactivation remains limited or inconsistent.

## 1. Introduction

Feline herpesvirus type 1 (FHV-1), an alphaherpesvirus that causes feline viral rhinotracheitis, primarily affects the upper respiratory tract and eyes during acute infection. Clinical manifestations include fever, rhinitis, conjunctivitis, and corneal disease [1,2,3,4]. Resolution of acute signs does not indicate viral clearance. FHV-1 can reach neural tissues through sensory nerves, with the trigeminal ganglia serving as the principal site of latency; latency-associated transcripts and viral DNA have also been detected in related neural or ocular tissues [5,6,7]. Stress or glucocorticoid-associated immunosuppression can trigger reactivation, recurrent ocular disease, and oronasal or conjunctival shedding [2,8,9]. FHV-1 is therefore both an acute disease and a population-level management problem sustained by lifelong latency, intermittent reactivation, and renewed shedding.

Vaccination is central to integrated FHV-1 control. Current products are mainly modified-live and inactivated vaccines, while more recent candidates include gene-deleted, replication-defective, and recombinant-vector platforms [1,10,11,12,13,14,15,16,17,18,19,20]. Vaccines may be administered parenterally, intranasally, or by combined routes. In practice, however, FHV-1 vaccination is intended primarily to reduce the occurrence or severity of clinical disease rather than to provide consistent sterilising immunity. Challenge studies have detected viral DNA or cultivable virus despite clinical improvement, and a temperature-sensitive intranasal vaccine can itself be shed and establish latency [17,20,21,22,23,24,25,26]. Neutralising antibody is an important immune measure, but no universally applicable one-to-one relation between FHV-1 antibody titre and complete clinical protection has been established [21,27]. Clinical disease mitigation must therefore be distinguished from sterilising immunity, and clinical protection, infection, viral shedding, latency, and reactivation should be evaluated as separate evidence domains.

Direct comparison of FHV-1 vaccine studies is difficult because interventions and models vary across several dimensions. Studies have evaluated monovalent or multivalent modified-live vaccines, inactivated vaccines, gene-deleted strains, and diverse recombinant candidates. Administration routes include subcutaneous, intramuscular, intranasal, oronasal, and combined regimens, while challenge strains, challenge doses, intervals from vaccination to challenge, and observation windows also differ [28,29,30,31,32]. Clinical outcomes have been reported as any clinical sign, fever, daily scores, cumulative scores, or disease-related removal. Virological outcomes include positivity at any time point, viral load, titre, peak load, or duration of shedding. Polymerase chain reaction (PCR) and quantitative PCR (qPCR) are more sensitive to low-level viral nucleic acid, whereas virus isolation detects cultivable virus; their analytical sensitivity and biological interpretation are not interchangeable [2,26,33]. Consequently, “vaccine efficacy” in this literature does not represent a single, consistently defined endpoint.

Evidence synthesis is further constrained by study size and reporting quality. Most in vivo feline experiments were small, and early reports provided little information on random sequence generation, allocation concealment, random housing, or blinding. Multi-arm designs and shared controls were common, and some studies administered feline calicivirus (FCV) and FHV-1 challenges sequentially, increasing the risks of duplicate weighting and incorrect attribution. Controlled challenges in specific-pathogen-free (SPF) cats assess efficacy under standardised conditions, but challenge dose, housing, and host background do not fully represent natural exposure. Field studies are instead susceptible to confounding by previous infection, vaccination history, and population setting [32,34]. Clinical signs, scores, fever, post-vaccination reactions, and virological outcomes have sometimes been interpreted together, while the structure of shedding, immunogenicity, latency, and reactivation data often precludes direct meta-analysis. Frameworks for animal-study risk of bias and reporting help identify these limitations, but FHV-1 vaccine evidence has not previously been integrated with consistent treatment of outcome definitions, study identity, shared controls, bias, and poolability [35,36].

We therefore conducted a systematic review of the effects of FHV-1 vaccination on clinical disease, viral shedding, immunogenicity, post-vaccination reactions, latency, and reactivation in cats. Exploratory meta-analysis was restricted to outcomes for which study definitions, control identity, observation windows, and data structures were sufficiently comparable. We also assessed risk of bias in animal studies, used prespecified sensitivity analyses to examine the robustness of quantitative findings to design and bias, and applied structured narrative synthesis to evidence that could not reasonably be pooled. This approach was intended to define both the conclusions supported by the current evidence and their principal uncertainties.

## 2. Materials and Methods

### 2.1. Search Strategy and Eligibility Criteria

PubMed, Web of Science, and the China National Knowledge Infrastructure (CNKI) were searched from database inception to 20 May 2026. The searches identified 172 records in PubMed, 130 in Web of Science, and 25 in CNKI. Title and abstract screening was followed by full-text eligibility assessment. Two reviewers independently screened titles and abstracts and assessed full-text eligibility.

The population was domestic cats. Eligible interventions were FHV-1 vaccines or multivalent vaccines containing an FHV-1 component. Comparators were unvaccinated, placebo, or other interpretable control groups. Eligible outcomes included FHV-1-related clinical disease, viral shedding or load, immunogenicity, post-vaccination reactions, safety, latency, or reactivation. Original controlled in vivo studies in cats were eligible. We excluded in vitro and non-feline studies; studies of antiviral drugs, natural products, or therapeutic antibodies; studies without a control group or an FHV-1 vaccine component; reviews; case reports; conference abstracts; patents; editorials; expert opinions; and duplicate datasets. Healthy, unchallenged mock groups were not used as vaccine-efficacy comparators.

### 2.2. Data Extraction

We extracted country, animal characteristics, sample size, baseline status, vaccine platform, antigen or strain, dose, route, schedule, comparator, challenge strain, challenge dose and route, interval from vaccination to challenge, observation window, outcome definition, event counts or continuous data, and safety information. Each vaccine arm was recorded separately. For multivalent vaccines, only FHV-1-related outcomes were extracted.

Reports, independent studies, animal cohorts, and comparisons were mapped hierarchically. Sussman et al. [25] was treated as a companion report of an earlier efficacy experiment: it did not add an independent study, cohort, or quantitative weight, but it contributed information on latency and reactivation. Field serology in Wang et al. [37] and in the Wu et al. [30] triple-inactivated-vaccine report may have included overlapping animals. These reports were therefore not pooled as two fully independent field cohorts.

### 2.3. Outcome Definitions and Hierarchy

The primary clinical outcome was at least one FHV-1-associated clinical sign after challenge. Key secondary clinical outcomes were fever after FHV-1 challenge, disease-related removal after FHV-1 challenge, and cumulative clinical score after FHV-1 challenge. Reactogenicity outcomes included at least one sneezing episode after vaccination and before challenge, together with other local or systemic reactions. Virological outcomes comprised shedding positivity, amount of shedding, peak viral load, and duration of shedding. Immunological outcomes comprised neutralising antibody, seroconversion, antibody titre, and other humoral measures. Latency-related outcomes comprised latent viral load, reactivation, and renewed shedding. Any post-challenge clinical score greater than zero was treated as an exploratory supplementary outcome.

### 2.4. Multi-Arm Studies and Shared Controls

Clinically and methodologically comparable intervention arms were combined where appropriate, and each shared control was used only once within an analysis. Original arm-level data were retained in the frozen data layer; shared controls were not duplicated. In the primary analysis, the three Tang et al. [17] gene-deletion constructs (ΔgI/gE, ΔTK, and ΔgI/gE/TK) were combined into one intervention group and compared with their shared control. The two commercial vaccine arms in Povey et al. [38] were similarly combined for the cumulative-score analysis. Post-vaccination sneezing was stratified into parenteral-only schedules and schedules containing an intranasal component, with no pooling across routes. The efficacy comparison for Wu et al. [21] was restricted to the monovalent FHV-1 strain 64 modified-live vaccine and the unvaccinated FHV-1-challenged control. The unchallenged mock group was excluded from effect estimation.

### 2.5. Risk-of-Bias Assessment

The Systematic Review Centre for Laboratory Animal Experimentation (SYRCLE) tool was used to assess ten domains: random sequence generation, baseline characteristics, allocation concealment, random housing, blinding of caregivers and investigators, random outcome assessment, blinding of outcome assessors, completeness of outcome data, selective reporting, and other bias. Each domain was judged Low, Unclear, or High [35]. For core outcomes included in quantitative synthesis, certainty of evidence was evaluated using the Grading of Recommendations Assessment, Development and Evaluation (GRADE) domains of risk of bias, inconsistency, indirectness, imprecision, and publication bias. Controlled animal challenge experiments began at high certainty as experimental evidence and were downgraded where warranted across these domains. Two reviewers independently assessed risk of bias.

### 2.6. Meta-Analysis

Binary outcomes were expressed as risk ratios (RRs), and continuous outcomes as mean differences (MDs) or small-sample-corrected Hedges’ *g*, each with a 95% confidence interval (CI). Common-effect estimates used the Mantel–Haenszel method without a continuity correction. Random-effects estimates used inverse-variance weighting, with between-study variance estimated by restricted maximum likelihood (REML). Heterogeneity was described using Q, τ^2^, and I^2^. Hartung–Knapp intervals were examined only as an additional interval check. An evidence matrix was first constructed by outcome hierarchy, study design, vaccine platform, administration route, and challenge model. Quantitative synthesis was undertaken only when outcome definitions, control identities, observation windows, and data formats supported a common effect scale; all other studies were retained in the structured narrative synthesis. Reports, studies, and animal cohorts were counted separately. Statistical analyses were performed in R version 4.4.3 using the metafor package version 5.0.1; figures were prepared using Python version 3.9.6 and Matplotlib version 3.9.4.

### 2.7. Reporting Guideline and Registration

This systematic review was conducted and reported in accordance with the Preferred Reporting Items for Systematic Reviews and Meta-Analyses (PRISMA) 2020 statement. The Open Science Framework (OSF) project record is available at https://doi.org/10.17605/OSF.IO/RFGU3 (accessed on 23 September 2026). The completed PRISMA 2020 checklist is provided as a Supplementary Table S1.

## 3. Results

### 3.1. Study Selection

**Figure 1:**
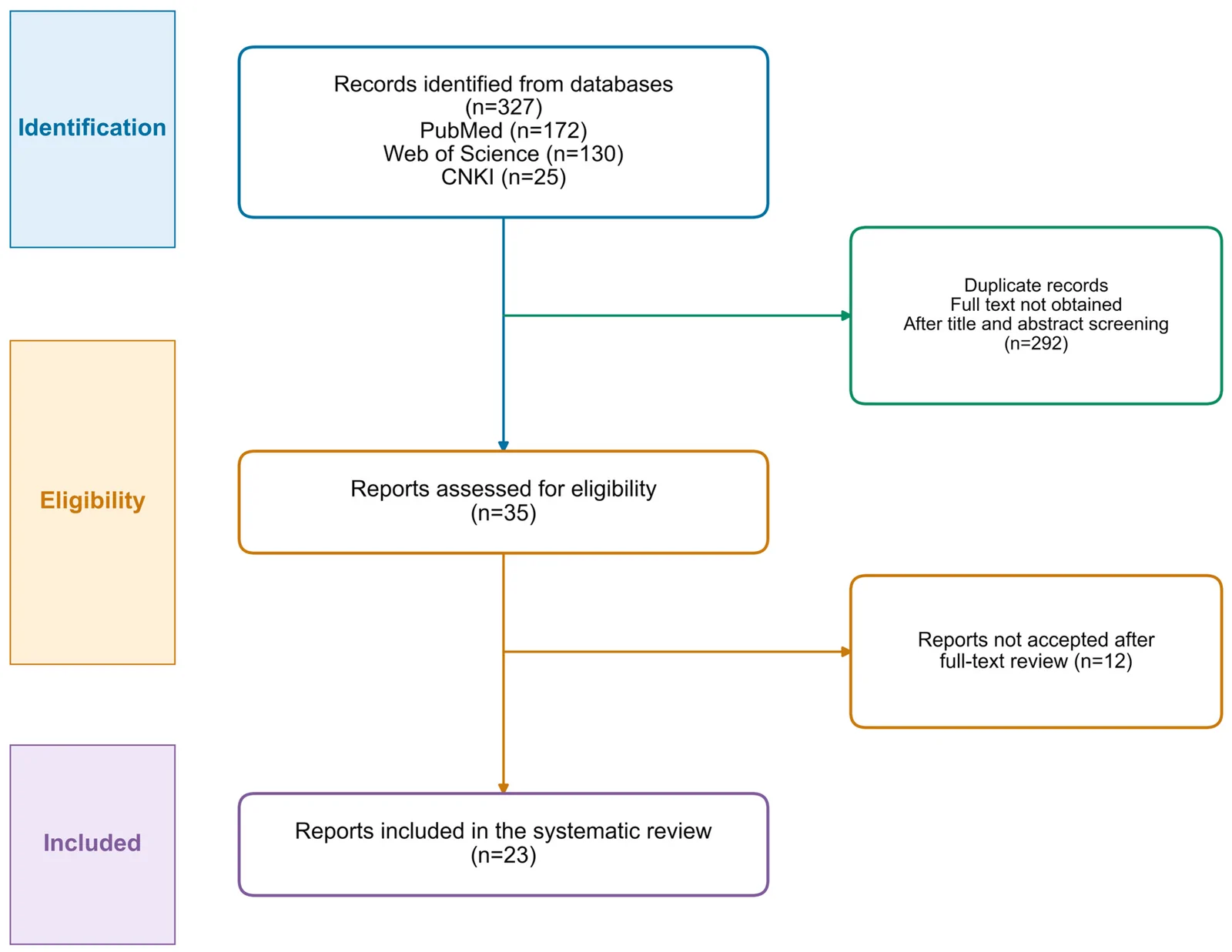
Study-selection flow diagram. The searches identified 172 records in PubMed, 130 in Web of Science, and 25 in CNKI. The 292 records not proceeding to full-text eligibility assessment comprised duplicate records, reports not obtained, and exclusions after title and abstract screening; separate category counts were not reported. Thirty-five full-text reports were assessed, and 12 were excluded. Twenty-three reports were included in the systematic review. Blue, orange, and purple denote identification, eligibility, and inclusion, respectively; arrows indi-cate the screening flow.

The searches identified 327 records across PubMed, Web of Science, and CNKI. A total of 292 records did not proceed to full-text eligibility assessment; this combined count included duplicate records, reports not obtained, and exclusions after title and abstract screening. Separate counts for these categories and the post-deduplication screening denominator were not reported. Thirty-five full-text reports were assessed for eligibility, 12 were excluded, and 23 reports were included (Figure 1). Sussman et al. [25] was a companion report and did not increase the number of independent studies or animal cohorts.

### 3.2. Characteristics of Included Studies

The 23 reports were published between 1977 and 2026 and originated from China, the United States, Canada, Japan, and several European countries. Most animals were healthy, SPF, or baseline-seronegative kittens, although the evidence also included feral cats vaccinated at neutering and large field-serology samples [37,39]. Experimental studies were generally small, commonly with 2–10 cats per group.

The evaluated vaccines comprised commercial multivalent modified-live or inactivated products, a monovalent FHV-1 modified-live vaccine (MLV), gI/gE/TK and other gene-deleted strains, a replication-defective FHV-1 vector, and recombinant vectors expressing antigens from feline panleukopenia virus (FPV), FCV, rabies virus, or granulocyte colony-stimulating factor (G-CSF) [12,13,14,15,16,17,18,19,20,21,24,28,30,31]. Administration routes included subcutaneous, intramuscular, intranasal, oronasal, and combined intranasal–parenteral schedules. Fifteen reports provided interpretable clinical challenge evidence, whereas seven mainly contributed immunogenicity, compatibility, or field-serology data without FHV-1 challenge. The Sussman companion report specifically contributed observations on latent viral load and reactivation [25]. Included-report characteristics are summarised in Table 1.

### 3.3. Risk of Bias

**Figure 2:**
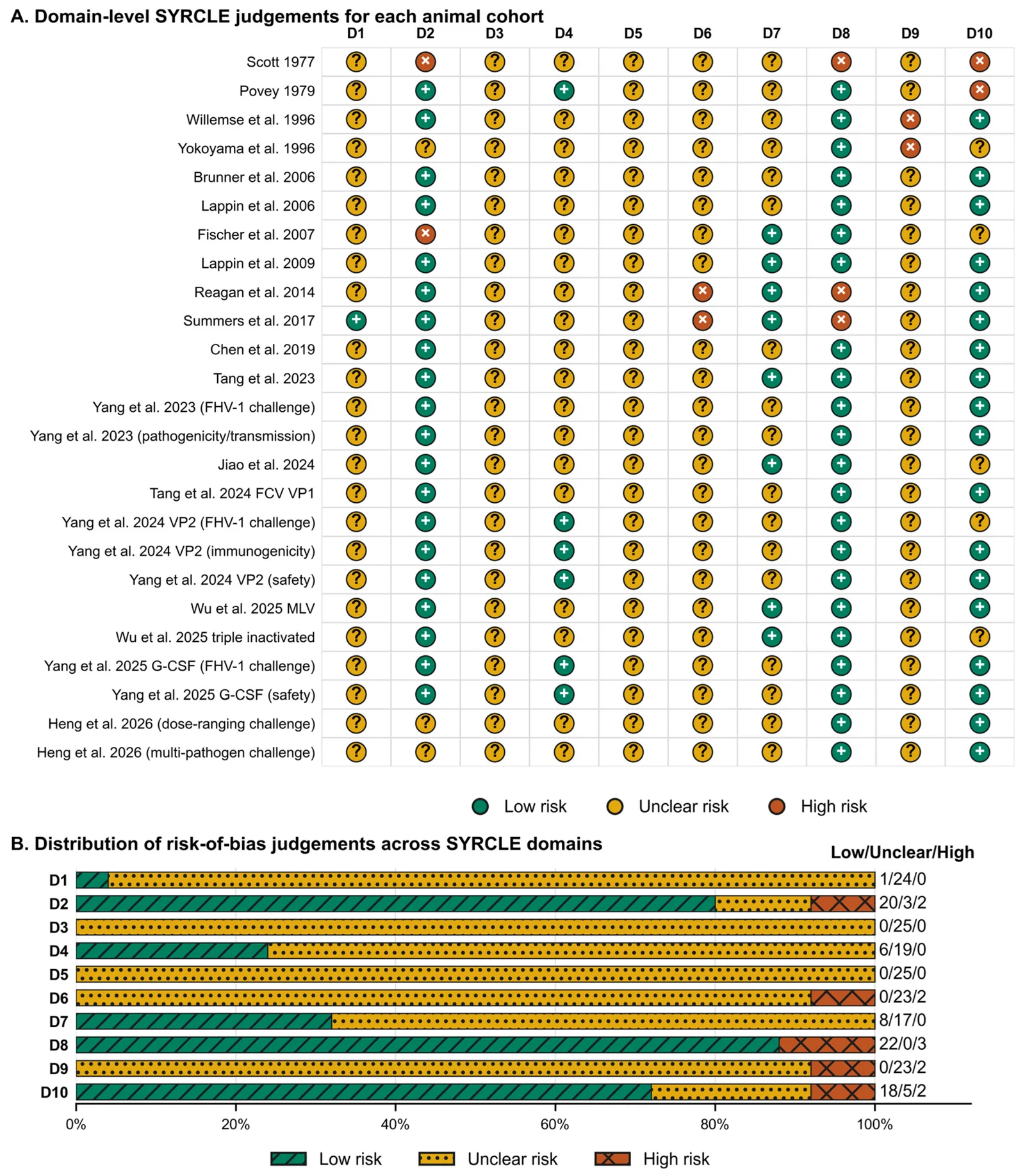
SYRCLE risk-of-bias assessment of included animal studies. Panel (**A**) presents domain-level judgements for each of the 25 animal cohorts; panel (**B**) summarises the distribution of Low, Unclear, and High judgements. D1, random sequence generation; D2, baseline characteristics; D3, allocation concealment; D4, random housing; D5, blinding of caregivers and investigators; D6, random outcome assessment; D7, blinding of outcome assessors; D8, completeness of outcome data; D9, selective reporting; D10, other bias. Green circles with “+”, yellow circles with “?”, and brown circles with “×” denote Low, Unclear, and High risk, respectively; the corresponding bar patterns indicate the same categories [12,14,15,16,17,18,19,20,21,22,23,24,28,29,30,31,32,39,40,41]. Assessments used the SYRCLE tool [35].

Risk of bias was assessed in 25 independent animal cohorts from 20 independent animal studies. The principal limitations were insufficient reporting of random sequence generation (D1: Low 1/Unclear 24/High 0) and no Low judgements for allocation concealment (D3: 0/25/0). Blinding of caregivers and investigators was universally Unclear (D5: 0/25/0), while random outcome assessment had limited reporting or explicit High judgements (D6: 0/23/2). Completeness of outcome data was comparatively well reported (D8: 22/0/3), whereas selective reporting (D9: 0/23/2) and other bias (D10: 18/5/2) remained concerns (Figure 2; Table 2).

### 3.4. Quantitative Synthesis of Post-Challenge Clinical Disease Outcomes

#### 3.4.1. At Least One FHV-1-Associated Clinical Sign After Challenge

**Figure 3:**
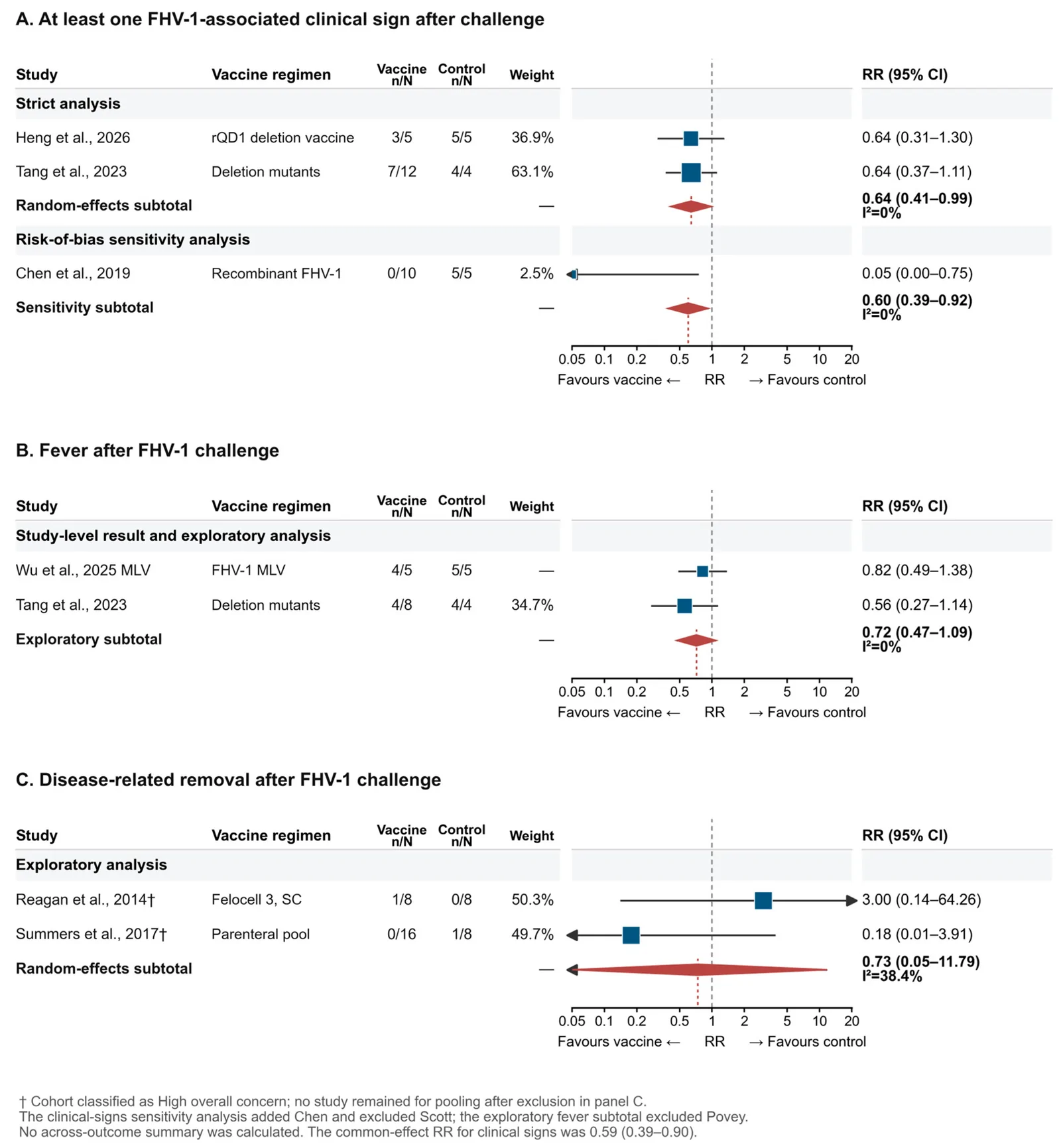
Effect of FHV-1 vaccination on clinical disease outcomes after challenge. Panel (**A**) shows at least one FHV-1-associated clinical sign after challenge, panel (**B**) shows fever after FHV-1 challenge, and panel (**C**) shows disease-related removal after FHV-1 challenge. Distinct outcomes are displayed separately; no overall effect across outcomes was calculated. RR values below 1 favour vaccination. CI, confidence interval; RR, risk ratio. Blue squares represent study-level estimates, with areas proportional to displayed weights where applicable; horizontal lines show 95% CIs. Red diamonds show pooled estimates and their CIs. Grey dashed lines mark the null effect; red dashed lines mark pooled estimates. Arrowheads indicate CIs extending beyond the plotted range; † identifies cohorts classified as High overall concern. n/N, animals with the event/animals analysed; I^2^, statistical heterogeneity. Source studies: [12,15,17,21,22,29].

The primary analysis included only Heng 2026 [12] and Tang 2023 [17] (*k* = 2; 17 vaccinated and nine control animals). The random-effects RR was 0.64 (95% CI 0.41–0.99), and the common-effect RR was 0.59 (95% CI 0.39–0.90). The three Tang et al. [17] constructs (ΔgI/gE, 2/4; ΔTK, 4/4; and ΔgI/gE/TK, 1/4) were combined according to the prespecified rule into one intervention group (7/12) and compared with the shared control (4/4), which was counted once. Because protection differed among the constructs, with no apparent protection in the ΔTK arm, the combined estimate represents the average effect of the candidate gene-deleted vaccine group. The prespecified risk-of-bias sensitivity analysis used an expanded evidence set that also included Chen et al. [15] and Scott [40]. After exclusion of Scott because of High overall concern, the analysis comprised Heng, Tang, and Chen (*k* = 3; 27 vaccinated and 14 control animals) and yielded RR 0.60 (95% CI 0.39–0.92), consistent in direction with the primary analysis [12,15,17]. The Scott comparison was 5/18 versus 5/8. Of 20 vaccinated cats, two with pre-existing FHV-1-neutralising antibody at vaccination were excluded, leaving 18; of ten contact controls, two died during the preceding FCV-challenge phase, leaving eight. Given the small numbers of studies and animals, these findings should be regarded as directional evidence. The clinical-sign estimates are shown in Figure 3A.

#### 3.4.2. Fever After FHV-1 Challenge

In the Wu 2025 study of the monovalent FHV-1 strain 64 MLV versus an unvaccinated challenged control, the study-level RR was 0.82 (95% CI 0.49–1.38) [21]. After exclusion of Povey, which used sequential FCV/FHV-1 challenge and was classified as High overall concern, the prespecified exploratory analysis comprised Wu and Tang (*k* = 2; 13 vaccinated and nine control animals) and yielded RR 0.72 (95% CI 0.47–1.09) [17]. Povey nevertheless reported the strongest directional signal for protection against fever in the evidence set: events occurred in 0/8 and 2/8 cats in the two vaccine arms and 8/8 controls [38]. Inclusion of Povey would make an exploratory pooled estimate more favourable to vaccination, but the sequential-challenge design and high risk of bias preclude treating it as a reliable estimate. The confidence intervals were compatible with both lower and higher risk and do not establish either benefit or absence of effect. Fever estimates are shown in Figure 3B.

#### 3.4.3. Disease-Related Removal After FHV-1 Challenge

The exploratory random-effects estimate from Reagan and Summers was RR 0.73 (95% CI 0.05–11.79) [22,29]. Both cohorts were classified as High overall concern, and no study remained after their exclusion. Events were sparse, and the interval was extremely wide, leaving the estimate highly imprecise. Disease-related removal estimates are shown in Figure 3C.

#### 3.4.4. Cumulative Clinical Score After FHV-1 Challenge

**Figure 4:**
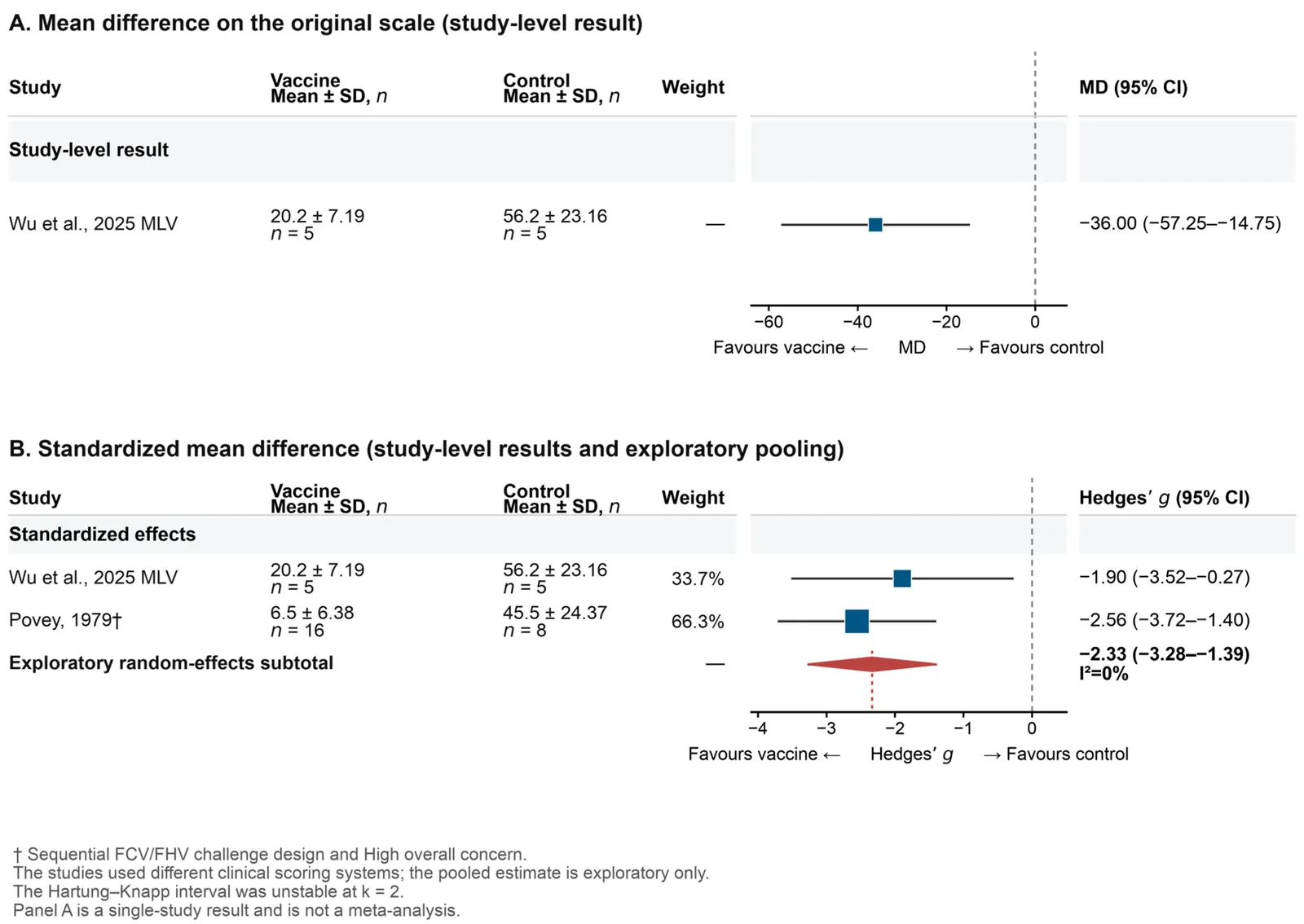
Effect of FHV-1 vaccination on cumulative clinical score after challenge. Panel (**A**) shows the Wu study-level mean difference (MD) on the original scale. Panel (**B**) shows study-level Hedges’ *g* estimates and the exploratory random-effects synthesis of Wu and Povey. MD and Hedges’ *g* are different effect measures and were not pooled together. The standardised synthesis is exploratory because only two small studies used different scoring systems, and Povey used a sequential FCV/FHV-1 challenge. CI, confidence interval. Blue squares represent study-level estimates, with areas proportional to displayed weights where applicable; horizontal lines show 95% CIs. Red diamonds show pooled estimates and their CIs. Grey dashed lines mark the null effect; red dashed lines mark pooled estimates. The null effect is zero. † denotes the sequential-challenge study classified as High overall concern; SD, standard deviation; I^2^, statistical heterogeneity. Source studies: [21,38].

In Wu, the MD between vaccinated and control animals was −36.00 (95% CI −57.25 to −14.75), with a corresponding Hedges’ *g* of −1.90 (95% CI −3.52 to −0.27) [21]. Wu and Povey used different scoring systems; after standardisation, their exploratory pooled Hedges’ *g* was −2.33 (95% CI −3.28 to −1.39; *k* = 2; 21 vaccinated and 13 control animals) [21,38]. The two Povey vaccine arms (Convac and FVR-C-P; eight cats each) were combined according to the prespecified rule and compared with the shared control of eight cats, which was counted once. Standardised mean differences can be upwardly biassed with only 5–16 animals per group, and effect sizes of this magnitude should be interpreted directionally. Povey used sequential FCV/FHV-1 challenge and was classified as High overall concern; only Wu remained after its exclusion. The Hartung–Knapp interval was unstable at *k* = 2 and therefore did not independently determine the interpretation. Cumulative-score estimates are shown in Figure 4; the main quantitative findings are summarised in Table 3.

### 3.5. Viral Shedding and Virological Outcomes

Fifteen reports provided information on shedding after acute challenge, viral load, virus isolation, or detection of vaccine virus after administration [12,13,14,17,19,20,21,22,23,24,29,30,31,38,40]. Assays included culture or virus isolation, qPCR or reverse-transcription qPCR (RT-qPCR) of nasal or ocular swabs, time-specific FHV-1/glyceraldehyde-3-phosphate dehydrogenase (GAPDH) ratios, titres expressed as log_10_ 50% tissue culture infectious dose (TCID_50_)/mL, and viral loads in tissue. Outcomes ranged from positivity at any time point to geometric means, peaks, and complete time series; sampling frequency and observation windows also varied.

The direction of effect in Heng, Tang 2025, Yang VP2, Reagan, Lappin 2006, the gene-deletion studies, and the G-CSF recombinant study generally indicated lower nasal or ocular viral loads or reduced shedding at selected time points in vaccinated animals [12,13,14,17,19,20,22,23]. Willemse reported mean post-challenge shedding of 0.6–1.8 log_10_ TCID_50_/mL across vaccination-route or strain groups, compared with 2.6 log_10_ TCID_50_/mL in controls [24]. Tang 2023 detected no virus in the gI/gE-deletion or triple-deletion groups, whereas the TK-deletion group reached a peak of approximately 10^1.5^ TCID_50_/mL and became negative by day 8 [17]. In the Wu monovalent MLV study, viral DNA appeared later, and the group-level duration was shorter in vaccinated animals, while controls remained positive for approximately 2 weeks [21].

Findings were not uniform. Summers found no clear group difference across its qPCR time series [29]. Lappin 2006 observed lower shedding only in selected groups on day 6, with no consistent difference at other time points [23]. Povey and Scott reported virus-recovery data, but sequential FCV/FHV-1 challenge, changing denominators, and older reporting formats limited direct synthesis [38,40]. In a preliminary study without FHV-1 challenge, Yokoyama reported detectable nasal recombinant virus in only one vaccinated cat and did not present the underlying data [31].

A single meta-analysis was not justified because shedding positivity, viral load, and duration of shedding are distinct outcomes, and assays and quantification limits differed. Sampling days and observation windows were inconsistent, some studies reported only figures or group-level descriptions, and several designs involved complex control or shared-control structures.

### 3.6. Humoral Immune and Serological Responses

Twenty reports provided interpretable evidence on FHV-1 neutralising antibody, seroconversion, antibody titres, or other humoral outcomes [12,13,14,15,16,17,18,19,20,21,22,28,29,30,31,37,38,39,40,41]. Most experimental modified-live, gene-deleted, or recombinant-vector vaccines elicited FHV-1-neutralising or gB-specific antibody after repeated immunisation. Some studies also reported interferon beta (IFN-β), cytokines, or lymphocyte subsets, but these data were usually presented only in figures, without extractable variances or complete individual-level values [12,13,14,15,16,17,19,20,21].

Studies of commercial vaccines and administration routes showed heterogeneous serological responses. In Lappin 2009, few cats met the laboratory definition of high-titre FHV-1 seroconversion after a single intranasal or parenteral feline viral rhinotracheitis–calicivirus–panleukopenia (FVRCP) vaccine dose, although more cats in both groups had detectable antibody when a lower threshold was applied [41]. In Brunner, FHV-1 indirect fluorescent antibody titres increased at some time points after co-administration, but no FHV-1 challenge was performed, and the authors cautioned that serological responses do not substitute for protection endpoints [28]. In the Fischer field study, the number of cats with protective anti-FHV-1 titres increased from 5/32 to 26/32 in the inactivated-vaccine group but remained 8/29 before and after vaccination in the MLV group. Interpretation is confounded by baseline exposure, vaccination at neutering, and the field design [39].

Wang et al. analysed 4736 vaccinated domestic cats from 24 Chinese provinces and reported mean FHV-1-neutralising antibody titres of approximately 111.4–152.7 across dose-schedule groups. Seropositivity was reported only as rounded percentages, so event counts could not be reconstructed [37]. The Wu triple-inactivated-vaccine report also included 1818 valid field samples [30]. Because authors and sampling periods overlapped across the two reports and individual sources could not be verified, the studies were described in parallel and were not pooled as independent cohorts. Antibody responses were observed across multiple platforms, but an increase in antibody cannot by itself be interpreted as clinical protection, prevention of infection, or prevention of latency.

### 3.7. Post-Vaccination Reactions and Safety

**Figure 5:**
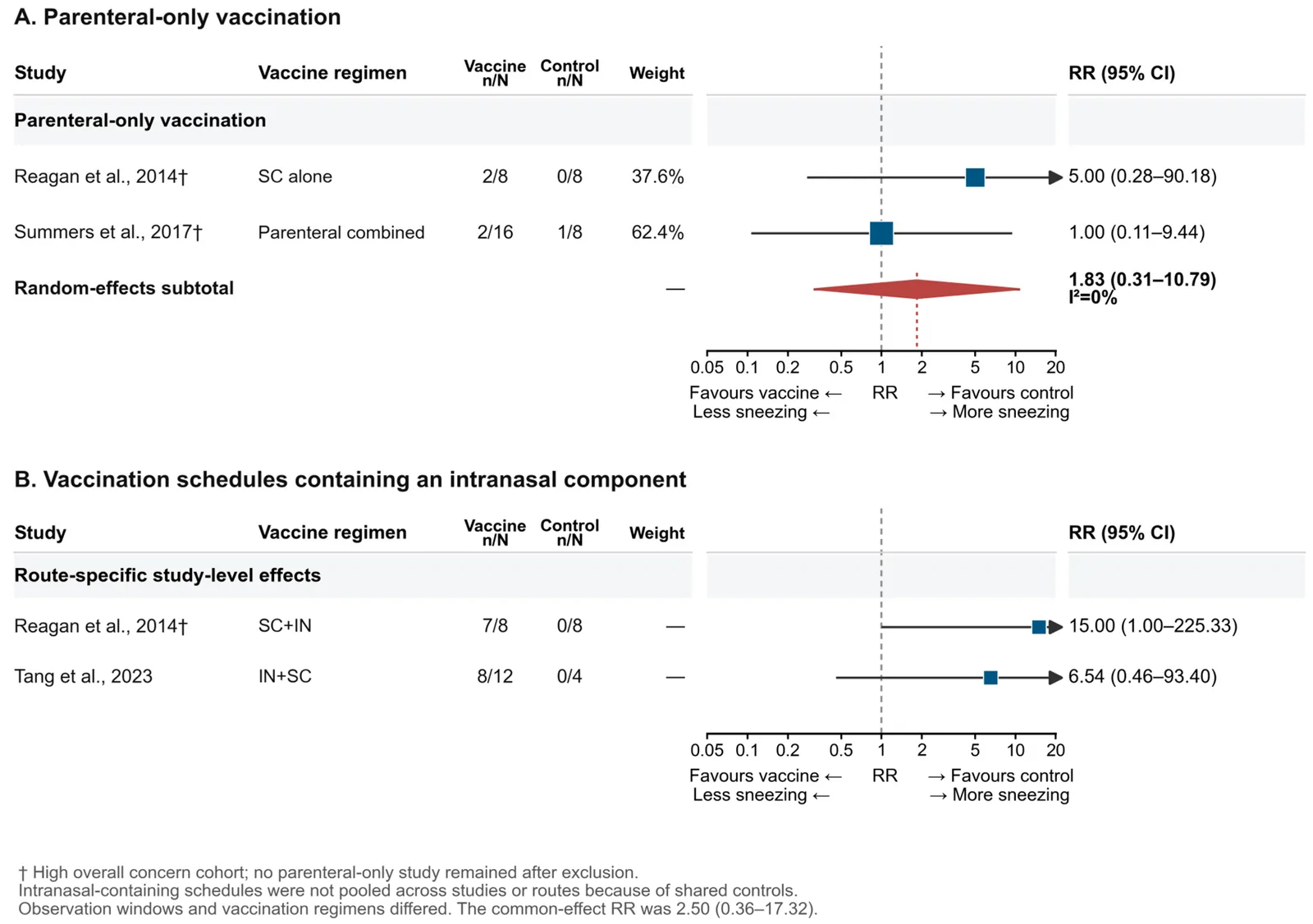
Sneezing after vaccination and before FHV-1 challenge. Panel (**A**) presents the exploratory random-effects synthesis of parenteral-only vaccination schedules. Panel (**B**) presents study-level effects for schedules containing an intranasal component; these were not pooled because routes and shared-control structures differed. RR values above 1 indicate more sneezing in vaccinated animals. CI, confidence interval; IN, intranasal; RR, risk ratio; SC, subcutaneous. Blue squares represent study-level estimates, with areas proportional to displayed weights where applicable; horizontal lines show 95% CIs. Red diamonds show pooled estimates and their CIs. Grey dashed lines mark the null effect; red dashed lines mark pooled estimates. The null effect is RR = 1. Arrowheads indicate CIs beyond the plotted range; † identifies cohorts classified as High overall concern. n/N, animals with sneezing/animals analysed; I^2^, statistical heterogeneity. Source studies: [17,22,29].

For at least one sneezing episode after vaccination and before challenge, the random-effects RR for parenteral-only schedules was 1.83 (95% CI 0.31–10.79). Schedules containing an intranasal component were presented as route-specific study-level effects: RR 15.00 (95% CI 1.00–225.33) in Reagan and RR 6.54 (95% CI 0.46–93.40) in Tang [17,22]. Outcomes were not pooled across routes (Figure 5). The intervals were extremely wide and did not establish the direction of risk. This was a reactogenicity outcome, not an outcome of protective efficacy.

Transient sneezing, nasal discharge, or detection of vaccine-strain DNA was reported more often after intranasal or combined-route vaccination. In Tang 2025, 1/4 kittens had mild sneezing for 2 days after the first intranasal dose; a similar reaction was shorter after the second combined intranasal and subcutaneous dose, and no pre-challenge fever occurred [13]. In the Reagan combined-route group, 7/8 cats sneezed, 3/8 coughed, and vaccine-strain DNA was detected in 6/8 [22]. In Lappin 2009, only one intranasally vaccinated cat developed mild nasal crusting on days 10 and 14; no other sneezing, coughing, or ocular discharge was reported [41].

Most parenteral studies reported no evident systemic reactions, although occasional local or transient reactions occurred. Brunner observed mild, transient local swelling in the feline leukaemia virus (FeLV)-adjuvanted group but no systemic signs [28]. In Summers, one kitten receiving inactivated vaccine developed fever, lethargy, and injection-site pain that resolved within 24 h [29]. Several safety studies of gene-deleted or recombinant vaccines reported normal temperature, body weight, and appetite without evident local erythema or pain [12,14,16,19,20]. Nasal or ocular discharge, fever, reduced appetite, or disease-related removal after challenge was not classified as a vaccine adverse event.

### 3.8. Latency and Reactivation

Latency and reactivation constituted the sparsest evidence domain. Sussman 1997 used animals from an earlier efficacy experiment and quantified wild-type FHV-1 DNA in the trigeminal ganglia, olfactory bulbs, and brainstem 8 weeks after challenge [25]. Latent load in trigeminal tissue was expressed as FHV-1 genome equivalents/µg tissue DNA (mean ± standard deviation [SD]). Values were 926 ± 1634 for the commercial vaccine, 7211 ± 10,799 for the gI/gE-deletion strain, 565 ± 576 for the parental strain, and 7276 ± 15,843 for the unvaccinated control. These data suggest that latent load may differ among vaccine strains, but each group contained only five cats, and dispersion was substantial.

Sussman reported no evidence of reactivation before euthanasia, but the study did not use a standardised glucocorticoid-induced reactivation protocol or provide long-term follow-up after repeated stress [25]. The available evidence is therefore insufficient to determine whether vaccination prevents the establishment of latency, lowers the probability of reactivation, or reduces renewed shedding. Some recent studies measured early viral loads in trigeminal or brain tissue, but their time points remained close to acute challenge and cannot be treated as long-term latency endpoints.

### 3.9. Evidence Patterns Across Vaccine Platforms and Administration Routes

Commercial modified-live and inactivated vaccines both had clinical or immunological evidence, but direct comparisons were few and heterogeneous. The Wu monovalent MLV showed favourable directions for clinical score and humoral and cellular immune responses [21]. In the Summers comparison of inactivated and modified-live parenteral vaccines, clinical, fever, and shedding outcomes did not establish a consistent advantage [29]. By contrast, the Fischer field study showed a more pronounced FHV-1 antibody response in the inactivated-vaccine group [39].

Gene-deleted and recombinant-vector studies generally reported lower clinical scores, reduced viral loads, or higher antibody responses [12,13,14,15,16,17,19,20]. Most, however, enrolled only 3–5 animals per group, presented outcomes mainly in figures, evaluated serial constructs from the same research groups, or used commercial comparators that were not fully equivalent. Multivalent vaccines also contained FCV, FPV, rabies virus, or other components. This review extracted only FHV-1-related outcomes and did not attribute protection against non-FHV challenges to the FHV-1 component.

Intranasal or oronasal schedules showed signals of earlier clinical mitigation or lower shedding in some studies [22,23,24], but transient sneezing or detection of vaccine virus was also more common. Parenteral schedules have a different reactogenicity background, and combined routes may affect both mucosal and systemic immunity. The evidence did not include enough direct, within-study randomised comparisons to establish that any vaccine platform or route was superior.

### 3.10. Heterogeneity in Challenge Models, Observation Windows, and Outcome Definitions

Challenge studies used different wild-type, laboratory, and field strains, at doses ranging from approximately 10^5^ to 10^6.5^ TCID_50_. Routes included intranasal, oronasal, and combined conjunctival–intranasal challenge. The interval from vaccination to challenge ranged from 2 to 6 days after a single intranasal dose to several weeks after completion of vaccination; Chen administered FHV-1 challenge approximately 180 days after vaccination [15,23]. Povey and Scott both used a preceding FCV challenge, which could affect later FHV-1 assessment through residual clinical status, immune activation, or removal of animals [38,40].

Observation windows and scoring systems were also inconsistent. Clinical outcomes included any sign, daily total score, cumulative score, days with fever, or disease-related removal; fever thresholds were not always defined. Virological outcomes included any positive sample, quantitative copy numbers, relative qPCR ratios, TCID_50_, or culture recovery, with differing sampling days. Some severely affected animals were removed before completion of follow-up, resulting in different denominators and interpretations for clinical scores and binary severe-disease outcomes. These differences directly prevented meta-analysis of many studies and were not merely general limitations raised in the discussion.

## 4. Discussion

This systematic review included 23 reports. Across the evidence, the most consistent potential effect of FHV-1 vaccination was mitigation of clinical disease after challenge rather than complete prevention of infection. The primary synthesis of clinical signs was restricted to two studies and 26 animals; the upper confidence limit for the random-effects estimate lay just below the null, and the risk-of-bias sensitivity analysis was consistent in direction. The small number of studies and animals nevertheless requires a cautious interpretation. Uncertainty was greater for fever, disease-related removal, and post-vaccination sneezing. Alongside the quantitative analyses, the structured synthesis identified extensive virological and immunological evidence, but differences in outcome level and study design precluded summarising it with a single overall effect.

Fewer clinical events or lower cumulative scores indicate whether vaccinated animals became ill less often or less severely; they do not directly indicate whether infection occurred. Challenge studies detected FHV-1 DNA or cultivable virus in vaccinated animals despite improvement in clinical outcomes [17,20,21,22,23,24,26,38]. Reviews and guidelines likewise characterise the principal protection offered by current vaccines as mitigation of disease rather than reliable prevention of infection [1,2]. We therefore treated clinical disease mitigation, prevention of infection, and sterilising immunity as distinct concepts.

Most recent recombinant or gene-deleted vaccine studies reported lower shedding or viral loads, but outcomes were often presented only in figures, limited to selected days, or expressed as relative measures. Findings from Summers, Lappin, and other studies indicated that reductions were not consistent at every time point [12,13,14,17,19,20,23,29]. PCR and qPCR can detect low-level viral nucleic acid, whereas virus isolation detects cultivable virus; their positivity rates and biological meanings are not interchangeable [2,26,33]. A reduction in viral DNA load therefore does not establish the disappearance of infectious virus or interruption of transmission. The studies also generally lacked standardised virus isolation, complete individual-level durations of shedding, and contact-transmission experiments. Cats can resume shedding after recovery or immunosuppression-induced reactivation [8,9], so short-term acute-phase outcomes do not fully characterise long-term transmission risk.

Twenty reports indicated that multiple platforms could elicit FHV-1-related antibodies. Neutralising antibody is an important immune measure, but no universally validated antibody threshold alone guarantees clinical protection against FHV-1 [27]. Immunogenicity-only studies lacked challenge endpoints, and field serology was affected by previous exposure, vaccination history, and sample selection. Even when antibody, cytokine, or lymphocyte measures were associated with clinical findings in challenge studies, correlation alone could not establish a causal mechanism. Direct FHV-1 evidence suggests that cellular measures may contribute to protection, but it is based mainly on small experiments [21]. Future studies should prespecify immunological, clinical, and virological endpoints in the same animals and report individual-level data suitable for assessing correlations and validating thresholds.

Favourable signals have been reported for modified-live, inactivated, gene-deleted, and recombinant-vector vaccines, but direct comparisons were sparse and serial studies may have shared experimental settings. Intranasal vaccination can induce local responses at the portal of infection and was associated with earlier clinical mitigation or lower shedding in some challenge experiments [1,22,23]. Transient sneezing, local discharge, or detection of vaccine virus also occurred after intranasal or combined-route vaccination [13,22,26,41]. Parenteral and combined routes have different reactogenicity and immunological contexts, and current guidelines recommend product- and risk-specific use [10,11]. Without adequate randomised head-to-head comparisons within the same studies, the available evidence does not establish the superiority of any vaccine platform or administration route.

Latency and reactivation were the least studied evidence domain, despite their biological importance. The trigeminal ganglia are the principal site of FHV-1 latency, and latency-associated transcripts or viral DNA can be detected without acute clinical signs [5,6,7]. Stress or glucocorticoid-associated immunosuppression can induce reactivation and renewed shedding [8,9]. The Sussman companion report showed that latent load was measurable and suggested that effects on wild-type latent load might differ among vaccine strains. Each group, however, contained only five cats; dispersion was large, and no standardised reactivation challenge was performed [25]. A temperature-sensitive intranasal vaccine likewise did not prevent establishment of latency [26]. Vaccination therefore cannot be assumed to prevent latency, and current evidence is insufficient to determine its stable effects on long-term latent load, the probability of reactivation, or renewed shedding.

Almost all cohorts had unclear reporting for at least one of random sequence generation, allocation concealment, or blinding. Risk of bias was especially consequential for disease-related removal and sneezing after parenteral vaccination because no studies remained after exclusion of cohorts classified as High overall concern. The SYRCLE tool and the Animal Research: Reporting of In Vivo Experiments (ARRIVE) guidelines 2.0 provide reproducible frameworks for assessing bias and improving reporting in animal intervention studies [35,36]. Controlled challenge experiments provide efficacy evidence under highly regulated conditions, but challenge doses, routes, and environments differ from natural exposure, and SPF kittens are not fully representative of pet or group-housed cats. Early dose–response experiments and studies of natural cat populations further support caution when extrapolating these findings [32,34]. Low statistical heterogeneity therefore does not eliminate clinical or methodological heterogeneity.

This review retained all 23 reports in the evidence matrix rather than restricting the review to studies eligible for meta-analysis. Reports, studies, cohorts, and comparisons were distinguished, allowing identification of a companion report, potentially overlapping field data, and shared controls. Formally defined outcomes separated clinical signs, clinical scores, fever, disease-related removal, and reactogenicity. Study-level estimates remained distinct from exploratory pooling, and risk-of-bias categories were linked to sensitivity analyses. The review also has limitations. Few studies and animals were available for pooling, and many outcomes were reported only in figures or as summaries without variance estimates. The evidence spans almost 50 years and includes highly heterogeneous challenge models, scoring systems, and outcome definitions. Field-serology reports may contain overlapping samples and did not undergo a design-matched risk-of-bias assessment. Standardised mean differences may be overestimated in small samples, so extreme estimates should be interpreted directionally. The number of studies was insufficient to assess publication bias.

## 5. Conclusions

Available animal studies suggest that FHV-1 vaccination may mitigate clinical disease after challenge, but this conclusion rests mainly on a few, small studies with incomplete methodological reporting. Evidence for fever, severe disease, viral shedding, post-vaccination reactions, latency, and reactivation remains limited or inconsistent. Future comparative experiments should prespecify clinical, virological, and long-term latency outcomes; use adequate randomisation, allocation concealment, and blinding; clearly report shared controls and animal flow; and provide individual-level data and complete time series in accordance with animal-research reporting standards [35,36]. Comparisons of vaccine platforms and administration routes should use the same challenge conditions and retain external validation under natural-exposure settings [10,11].

## Figures and Tables

**Table 1:** Characteristics of included reports.

Report	Country	Animals/Sample	Vaccine Platform	Route	FHV-1 Challenge	Principal Evidence Contribution
Heng et al. 2026 [12]	China	Dose-ranging experiment: high-dose arm *n* = 6 allocated (one cat euthanised before challenge); other dose arms *n* = 5 each; shared DMEM control *n* = 5. Trivalent experiment: vaccine and DMEM control *n* = 15 each; FHV-1 challenge subsets *n* = 5 each.	Replication-defective live FHV-1 Delta gL vector	Primary: IM; booster: IM	WT FHV-1 QD1	Clinical, virological, immunological, and safety/reactogenicity
Tang et al. 2025 [13]	China	Vaccinated *n* = 4, Control *n* = 4	Live attenuated recombinant FHV-1 vector vaccine/gene-engineered trivalent vaccine	Primary: IN booster: IN + SC	FHV WX19 strain	Clinical, virological, immunological, and safety/reactogenicity
Yang et al. 2024 [14]—VP2	China	FHV challenge arm *n* = 5; shared DMEM FHV challenge control *n* = 5	Live recombinant FHV-1 mutant; commercial modified live multivalent vaccine	Primary: SC; booster: NA	FHV-1 WH2020	Clinical, virological, immunological, and safety/reactogenicity
Brunner et al. 2006 [28]	Switzerland/UK	*n* = 10 per group; analysed *n* = 10	Commercial modified live multivalent vaccine with or without recombinant FeLV vaccine	Primary: SC; booster: SC	No FHV-1 challenge	Immunological and safety/reactogenicity
Wu et al. 2025 [21]—MLV	China	Vaccine *n* = 5; unvaccinated challenge control *n* = 5; mock medium unchallenged *n* = 3	Modified live FHV-1 vaccine	Primary: SC; booster: SC	FHV-1 strain 64	Clinical, virological, immunological, and safety/reactogenicity
Reagan et al. 2014 [22]	USA	Vaccine arm *n* = 8; unvaccinated control *n* = 8; one SC kitten removed day 9 post-challenge for later analyses	Commercial modified live FVRCP and/or IN FHV/FCV vaccine	Primary: SC + IN; booster: NA; Primary: SC; booster: NA	USDA FHV-1 Challenge Lot 96-13	Clinical, virological, immunological, and safety/reactogenicity
Summers et al. 2017 [29]	USA	*n* = 8 per group; control *n* = 8; one control removed day 10 post-challenge	Commercial inactivated or modified live parenteral FVRCP vaccine	Primary: SC; booster: NA	USDA Feline Rhinotracheitis Challenge Lot 96-13	Clinical, virological, immunological, and safety/reactogenicity
Lappin et al. 2006 [23]	USA	Vaccine arm *n* = 10; shared unvaccinated control *n* = 10	Commercial modified live intranasal FVRCP vaccine	Primary: IN; booster: NA	Feline Rhinotracheitis Challenge Virus SGE Lot 96-13	Clinical, virological, and safety/reactogenicity
Scott 1977 [40]	USA	20 vaccinated; 10 unvaccinated contact controls	Attenuated combined respiratory disease vaccine	Primary: IM; booster: IM	FVR 1101	Clinical, virological, immunological, and safety/reactogenicity
Chen et al. 2019 [15]	China/USA	*n* = 5 per relevant arm; unvaccinated FHV challenge control *n* = 5	Recombinant FHV-1 vectored rabies vaccine; commercial inactivated multivalent vaccine	Primary: IN; booster: IN; Primary: IN; booster: NA; Primary: SC; booster: SC	FHV-Z field isolate	Clinical, immunological, and safety/reactogenicity
Lappin et al. 2009 [41]	USA	*n* = 5 per group; analysed *n* = 5	Commercial modified live FVRCP vaccine	Primary: IN; booster: NA; Primary: SC; booster: NA	No FHV-1 challenge	Immunological and safety/reactogenicity
Jiao et al. 2024 [16]	China	Inactivated SRV9-FHV-gB *n* = 3; PBS control *n* = 2	Inactivated recombinant rabies virus vector expressing FHV-1 gB	Primary: SC; booster: SC; second booster: SC	No FHV-1 challenge	Immunological and safety/reactogenicity
Willemse et al. 1996 [24]	Netherlands/UK	*n* = 4; unvaccinated control *n* = 4; *n* = 3; unvaccinated control *n* = 4	Live attenuated/recombinant FHV-1 strain	Primary: oronasal; booster: NA; Primary: SC; booster: SC	Virulent FHV-1 SGE strain (NVSL, USA)	Clinical, virological, and safety/reactogenicity
Wu et al. 2025 [30]—triple inactivated	China	FHV cohort: vaccine *n* = 3, commercial comparator *n* = 3, challenged control *n* = 3, healthy unchallenged *n* = 2	Inactivated multivalent vaccine	Primary: SC; booster: SC	FHV-1 strain NR/64	Clinical, virological, immunological, and safety/reactogenicity
Tang et al. 2023 [17]	China	*n* = 4 per vaccine arm; unvaccinated control *n* = 4	Live FHV-1 gene-deleted mutant	Primary: IN; booster: IN + SC	Virulent FHV WX19	Clinical, virological, immunological, and safety/reactogenicity
Tang et al. 2024 [18]—FCV VP1	China	Recombinant vaccine *n* = 4; cell culture medium control *n* = 4	Live recombinant FHV-1 Delta gI/gE vector expressing FCV VP1	Primary: IN; booster: NA	No FHV-1 challenge	Immunological and safety/reactogenicity
Yokoyama et al. 1996 [31]	Japan	C7301dlTK-Cap *n* = 2; C7301dlTK vector control *n* = 1	Live recombinant TK-deficient FHV-1 expressing FCV capsid protein	Primary: IN + oral; booster: IN + oral	No FHV-1 challenge	Virological, immunological, and safety/reactogenicity
Yang et al. 2025—G-CSF [19]	China	FHV efficacy *n* = 3 per arm; DMEM control *n* = 3	Live recombinant FHV-1 mutant; Commercial modified live vaccine	Primary: SC; booster: NA	FHV-1 WH2020	Clinical, virological, immunological, and safety/reactogenicity
Fischer et al. 2007 [39]	USA	*n* = 32 allocated/analysed; *n* = 29 allocated/analysed	Commercial inactivated or modified live FVRCP-FeLV vaccine	Primary: SC; booster: NA	No FHV-1 challenge	Immunological and safety/reactogenicity
Yang et al. 2023 [20]	China	*n* = 5 per efficacy group; DMEM control *n* = 5	Live TK/gI/gE-deleted FHV-1 mutant; Commercial modified live trivalent vaccine	Primary: SC; booster: SC; second booster: SC	Highly virulent FHV-1 WH2020	Clinical, virological, immunological, and safety/reactogenicity
Povey 1979 [38]	Canada	*n* = 8 per vaccine arm; unvaccinated control *n* = 8	Commercial FVRCP vaccine	Primary: IM; booster: IM	FVR strain 605	Clinical, virological, immunological, and safety/reactogenicity
Sussman et al. 1997 [25]	USA	*n* = 5 per vaccine arm; unvaccinated control *n* = 5	Live FHV-1 vaccine/recombinant strain	Primary: SC; booster: SC	Virulent FVR-SGE field strain	Clinical, latency, and reactivation; companion report
Wang et al. 2025 [37]	China	One-dose schedule: analysed *n* = 107; two-dose schedule: analysed *n* = 3433; three-dose schedule: analysed *n* = 424; annual booster schedule: analysed *n* = 409.	Commercial inactivated multivalent vaccine	NR	No FHV-1 challenge	Immunological

Abbreviations: DMEM, Dulbecco’s modified Eagle medium; FCV, feline calicivirus; FeLV, feline leukaemia virus; FHV-1, feline herpesvirus type 1 (also abbreviated FHV in source study labels); FVR, feline viral rhinotracheitis; FVRCP, feline viral rhinotracheitis–calicivirus–panleukopenia; G-CSF, granulocyte colony-stimulating factor; IM, intramuscular; IN, intranasal; MLV, modified-live vaccine; NA, not applicable; NR, not reported; PBS, phosphate-buffered saline; SC, subcutaneous; TK, thymidine kinase; WT, wild type. gB, gE, gI and gL denote glycoproteins B, E, I and L; VP1 and VP2 denote viral proteins 1 and 2. Strain and product names are retained as reported.

**Table 2:** Summary of SYRCLE risk-of-bias domains across 25 animal cohorts.

Domain	Name	Low	Unclear	High	Main Interpretation
D1	Random sequence generation	1	24	0	Predominantly insufficient reporting
D2	Baseline characteristics	20	3	2	Includes explicit high judgements
D3	Allocation concealment	0	25	0	Predominantly insufficient reporting
D4	Random housing	6	19	0	Predominantly insufficient reporting
D5	Blinding of caregivers/investigators	0	25	0	Predominantly insufficient reporting
D6	Random outcome assessment	0	23	2	Predominantly insufficient reporting
D7	Blinding of outcome assessors	8	17	0	Predominantly insufficient reporting
D8	Completeness of outcome data	22	0	3	Includes explicit high judgements
D9	Selective reporting	0	23	2	Predominantly insufficient reporting
D10	Other bias	18	5	2	Includes explicit high judgements

**Table 3:** Main quantitative findings.

Outcome	Analysis	Studies	*k*; Vaccine/Control	Measure	Effect	95% CI	Interpretation
At least one FHV-1-associated clinical sign after challenge	Primary random-effects	Heng + Tang	2; 17/9	RR	0.64	0.41–0.99	Directional evidence
At least one FHV-1-associated clinical sign after challenge	Common-effect	Heng + Tang	2; 17/9	RR	0.59	0.39–0.90	Small *k*; supplementary only
At least one FHV-1-associated clinical sign after challenge	Excluding Scott	Heng + Tang + Chen	3; 27/14	RR	0.60	0.39–0.92	Risk-of-bias sensitivity analysis
Fever after FHV-1 challenge	Study-level result	Wu MLV	1; 5/5	RR	0.82	0.49–1.38	Highly imprecise
Fever after FHV-1 challenge	Excluding Povey	Wu + Tang	2; 13/9	RR	0.72	0.47–1.09	Heterogeneous definitions/models
At least one sneezing episode after vaccination and before challenge	Parenteral random-effects	Reagan + Summers	2; 24/16	RR	1.83	0.31–10.79	Reactogenicity; no pooling across routes
Disease-related removal after FHV-1 challenge	Exploratory random-effects	Reagan + Summers	2; 24/16	RR	0.73	0.05–11.79	All High overall concern
Cumulative clinical score after FHV-1 challenge	Study-level result	Wu	1; 5/5	MD	−36.00	−57.25 to −14.75	Same scoring scale
Cumulative clinical score after FHV-1 challenge	Study-level standardised	Wu	1; 5/5	Hedges’ *g*	−1.90	−3.52 to −0.27	Small-sample correction
Cumulative clinical score after FHV-1 challenge	Exploratory standardised	Wu + Povey	2; 21/13	Hedges’ *g*	−2.33	−3.28 to −1.39	Sequential challenge; unstable HK interval

Abbreviations: CI, confidence interval; HK, Hartung–Knapp; *k*, number of studies; MD, mean difference; MLV, modified-live vaccine; RR, risk ratio. Vaccine/control values are numbers of animals; Hedges’ *g* is the small-sample-corrected standardised mean difference.

## Data Availability

All included studies are cited in the reference list.

## Supplementary Materials

The following supporting information can be downloaded at: https://www.mdpi.com/article/10.3390/v18101115/s1, Table S1: PRISMA 2020 checklist [42].
